# A Deep Learning Approach to Lunar Rover Global Path Planning Using Environmental Constraints and the Rover Internal Resource Status

**DOI:** 10.3390/s24030844

**Published:** 2024-01-28

**Authors:** Toshiki Tanaka, Heidar Malki

**Affiliations:** 1 Department of Electrical and Computer Engineering, University of Houston, Houston, TX 77004, USA; 2 Department of Engineering Technology, University of Houston, Houston, TX 77004, USA

**Keywords:** path planning, planetary rover, lunar rover, reinforcement learning

## Abstract

This research proposes a novel approach to global path and resource planning for lunar rovers. The proposed method incorporates a range of constraints, including static, time-variant, and path-dependent factors related to environmental conditions and the rover’s internal resource status. These constraints are integrated into a grid map as a penalty function, and a reinforcement learning-based framework is employed to address the resource constrained shortest path problem (RCSP). Compared to existing approaches referenced in the literature, our proposed method enables the simultaneous consideration of a broader spectrum of constraints. This enhanced flexibility leads to improved path search optimality. To evaluate the performance of our approach, this research applied the proposed learning architecture to lunar rover path search problems, generated based on real lunar digital elevation data. The simulation results demonstrate that our architecture successfully identifies a rover path while consistently adhering to user-defined environmental and rover resource safety criteria across all positions and time epochs. Furthermore, the simulation results indicate that our approach surpasses conventional methods that solely rely on environmental constraints.

## 1. Introduction

The need for energy efficient operation in the space environment and specifically on the Moon is paramount to effective commercial and scientific missions in vast lunar/planetary surfaces. The techniques are categorized at the first level into global (offline) and local (online) path planning approaches [1]. Global path planning involves computing an initial path using available global map information, aiming to optimize a target metric such as the vehicle’s travel distance. Typically, this computation occurs in a mission operation center on the ground. The resultant data are then either employed by ground operators for manual operation or transmitted to space rovers for autonomous operation [2]. On the contrary, local path planning techniques are reactive in nature. They come into play to adjust the initially calculated path by the global planner in response to unforeseen situations, such as the sudden presence of obstacles. The local path planning is ideally conducted on onboard computers for increased autonomy. In this research, our focus is on the global path planning problem for lunar surface missions, especially in application to ’small’ lunar rovers where limited size and power capability impose extended environmental and resource constraints.

In the conventional global path planning for terrestrial applications, the primary goal is to achieve maximum speed for swift surface coverage. However, when addressing additional operational conditions, the concept of accelerated exploration does not necessarily equate to effective planning. A number of studies have been conducted on global path planning, employing different algorithms to address various environmental considerations: obstacle avoidance [3] (MDP); terramechanics [4,5] (Dijkstra), [6] (Reinforcement learning); sun-synchronous motion [7] (A*), [8] (Multi-speed spatiotemporal A*); terramechanics and power generation [9] (A*), [10] (Reinforcement learning); thermal condition, power generation, and terramechanics [11] (Dijkstra); uncertainty of the information [12] (RRT*); and hazard risk and collision avoidance [13] (A*), [14] (MDP), [15] (A*). These studies emphasize the importance of carefully selecting mathematical models and algorithms based on the specific purpose and constraints to be taken into account in the path planning process.

In the context of lunar surface exploration mission scenarios involving a compact rover with resource constraints, careful consideration must be given to a range of pivotal design factors. One of the key factors is the rover terramechanical constraint. Due to size constraints, many small rovers are equipped with small wheels, limiting their ability to climb slopes [16]. Also, due to the limited battery capacity of small rovers, it is essential to find an energy-efficient path to avoid excessive power consumption [4,5]. Consequently, it is of high importance to search an optimal path from a terramechanical cost point of view.

Another important factor is an increased sensitivity to thermal and luminous conditions on the lunar surface. As the size of rovers decreases, thermal capacitance and battery size are reduced. As a result, small rovers will cause immediate change in temperature and battery status in accordance with local lunar surface temperature as well as the sun position, which constantly changes over the course of the mission period. Therefore, it is essential to control when to move (timings of relocation), as well as where to move (path), to circumvent the variation in thermal and luminous conditions the rover will encounter [7,8,9,10,11].

There are fundamentally two possible ways to consider thermal and power constraints in the path planning process. One option is to use extrinsic conditions, such as lunar surface temperature and luminous environmental conditions, to determine immediate traversal/untraversal areas, such as those described in [7,8,9,10,11]. In this research, this scenario is called *environment-based path search*. Depending on the temporal characteristic of environmental conditions, constraints become either time-variant or static. The other option is to directly consider the rover’s intrinsic status, such as the internal temperature and battery power, in determining possible paths, which has not been explored in the existing literature. In this research, this scenario is called *rover resource-based path search*. Theoretically, the rover resource-based path search will result in more flexible path selection, as the rover’s thermal and power system have a capacitance and a short period of exposure to a harsh environment, which may be avoided if the path search is performed based on the environmental conditions that can be circumvented. Therefore, using the rover resource status rather than environmental conditions for path planning can increase flexibility and optimality of the path search. Rover resource status is dependent on its previous status and, as a result, elicits path-dependent constraints. To perform the rover resource-based path search, the path planning framework must cope with not only static and time-variant constraints, but also path-dependent constraints.

Upon closely examining the most related work, Oikawa et al. [11] addressed thermal and power constraints within the path planning process by approximating them as time-fixed costs and solving the problem using Dijkstra’s algorithm. Another approach, as presented by Hu et al. [10], involved applying reinforcement learning to a graph after extracting slope and illumination features of the map of the moment. While these approaches offer a good approximation in relatively stable environments, the reliability of path search results diminishes when underlying assumptions are not upheld, necessitating a repeat of the path search. Essentially, these methods are not suitable when searching for an optimal path over an extended duration in time-variant environments. Otten et al. [7], Hu et al. [8], and Ji et al. [9] incorporated power constraints by integrating the time-variant lunar surface luminous condition through the expansion of the graph in the temporal direction (in other words, generating a 3D binary array composed of stacked 2D maps for each time step). They addressed this using either the A* algorithm or the multi-speed spatiotemporal A* algorithm. While these methods are efficient in solving their specific challenges, these approaches cannot account for path-dependent constraints. As a result, they cannot directly utilize the internal resource status of the rover in the path planning and the path searches they employ may lead to less economical paths.

To address the limitations of the existing work, this research proposes a reinforcement learning-based approach that can directly handle path-dependent constraints and, consequently, enables the rover resource-based path search. To the best of our knowledge, the integration of rover thermal and power resource considerations into the reinforcement learning-based path planning framework within the domain of lunar rover missions is new. The proposed approach is capable of incorporating static, time-variant, and path-dependent constraints onto a grid map using a penalty function. Subsequently, it utilizes a reinforcement learning framework to solve a resource-constrained shortest path problem (RCSP) over the generated map. Importantly, all constraints are simultaneously applied to a graph, eliminating the need for a hierarchical structure. This feature serves as a key enabler in comprehending the inter-relationships among constraints and path selection. Additionally, the proposed framework can also consider rover motion transition probability (the rover may go to locations not originally planned with a certain probability), which is critical to small rover systems due to the fact that their navigational sensor system may be limited by their size and power capacity. A comparison of the aforementioned global path planning methods is depicted in Table 1.

To summarize, the major advantages of the proposed method over the existing research are: (1) the proposed method can consider path-dependent constraints, which can produce more flexible path selections than the case of only using static and time-variant constraints; (2) the proposed method can deal with various types of constraints simultaneously, without relying on a hierarchical architecture, enabling understanding of inter-relationships between these constraints and path selection; and (3) the proposed method can consider rover motion transition probability during the path planning, which is critical for the case of small rovers whose navigational sensors are low performance.

The remainder of the paper is organized into six sections. Section 2 presents the problem formulation and proposed architecture. Section 3 provides detailed mathematical models. Section 4 summarizes numerical simulation details and results. In Section 5, a comparative analysis is presented. Section 6 offers further insights into the proposed architecture based on simulation results or additional simulations. Finally, Section 7 provides concluding remarks.

## 2. Methods

### 2.1. Problem Statement

This study addresses the challenge of identifying energy-constrained shortest paths, framing it as a RCSP problem. This problem falls within the realm of combinatorial optimization, specifically defined on a graph. The objective is to determine a feasible optimal path between two specified nodes while adhering to predefined constraints. In our case, constraints include static type (terrain slope) and path-dependent type (rover thermal and power status), whereas environmental inputs (heat flux and illumination on the moon) are time-variant. The planning is performed in multi-objective planning rather than minimum-time planning. Since environmental inputs are time-variant, the rover is permitted to wait (i.e., stay in the same position until the next time step) to avoid excessive heat input. As a result, the first-in-first-out (FIFO) property, which essentially states that delaying departure time can never result in earlier arrival, is violated.

### 2.2. Proposed Learning Architecture

**Figure 1:**
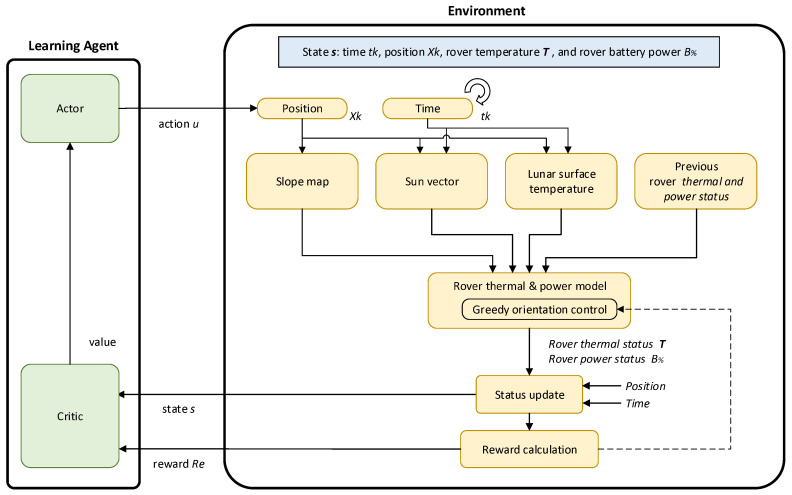
Proposed learning framework.

Figure 1 shows the architecture of the proposed path planning approach using reinforcement learning. Reinforcement learning is a learning-based method for optimal decision making and control. The agent acquires control profiles through the exploration of an environment using a trial-and-error process. *Deep Reinforcement Learning* (*DRL*) is the combination of reinforcement learning and deep learning [17]. It is also the most trending type of machine learning, because it can solve a wide range of complex decision-making problems that were previously out of reach. DRL has been applied to the path planning and control problems of mobile robots [18,19], unmanned aerial vehicles [20], and underwater robots [21]. In particular, this research utilizes the *Deep Q-Network* (*DQN*), which is a subset of DRL. It is a model-free, online, off-policy reinforcement learning method [22]. DQN combines Q-learning with deep neural networks, using a neural network to approximate the Q-function and enabling it to handle high-dimensional input spaces. DQN is a popular choice for discrete action spaces, and is also suitable for graph-based path-planning problems.

As the mathematical framework solved with DQN, the *Markov Decision Process* (MDP) was utilized. MDP is a mathematical model used to describe decision-making problems in situations where an agent interacts with an environment. MDP is characterized with a 4-tuple (*S*, *U*, $P_{a}$, $R_{e}$), where *S* is a set of states, with each state represented by s∈S; *U* is a set of actions, where each action is denoted as u∈U; $P_{a}$ is the state-transition function, which provides the probability of a transition between every pair of states given each action; and $R_{e}$ is a reward function that assigns a real value to each state/action pair. The solution to a MDP involves finding an optimal policy that maximizes the expected sum of rewards over time.

In this research, the state *s* and action *u* were defined using discrete variables. The state *s* was designed to incorporate the rover position ($X_{k}$), time ($t_{k}$), rover thermal status (T), and rover power status ($B_{\%}$), thus forming $s=\{X_{k},t_{k},T,B_{\%}\}$. By definition, state *s* was designed to satisfy the *Markov Property*, meaning that the current state can be determined solely by using the input to the current time step and its immediate previous state (i.e., a memoryless system). The reward function is defined in accordance with the *Markov reward process*, wherein the reward function provides a numerical score based on the state of the environment. Each element of both the state model and reward model is described in detail in Section 3.

### 2.3. Environment

The environment module was implemented over a graph; more particularly, a grid world map representing a rover mission scenario, where the rover explores a specific point on the lunar surface on a designated date and moves from a defined starting point to a predetermined goal. Details of the map structure are described in Section 3.1.

Depending on the rover’s location and time-epoch, the module determines the slope angle, sun vector, and lunar surface temperature, which will then be applied to the rover for the current time step. Subsequently, the rover’s thermal and power status of the time step is calculated, taking into account the determined slope angle, sun vector, lunar surface temperature, and the rover’s thermal and power status from the previous time step, using a designed rover thermal and power model. Finally, the reward value Re is computed based on the updated state *s*, which is then provided to the learning agent. Using these values, the learning agent determines a new action *u*, which dictates whether the rover should stay in its current position or relocate for the next time step. Detailed mathematical models for the sun vector and lunar surface temperature are provided in Section 3.2, while the rover thermal and power models are elaborated on in Section 3.3.

### 2.4. Limitations and Scope

The actual performance may vary based on the accuracy of the environment model and rover model. This research utilized realistic data to the best extent possible. For example, the slope data are derived from a real lunar digital elevation model with high accuracy. Moreover, it is well-established that the sun vector and lunar surface temperature can be accurately predicted using a mathematical model, given the absence of atmosphere and the low conductivity of the lunar surface.

However, it is conceivable that certain parameters related to the lunar surface, such as absorptivity and emissivity, may require calibration, especially in the presence of small topological features like craters, considering the age of the terrains.

The rover model is based on a real flight project [23], which has undergone calibration through a thermal balance test conducted in a vacuum chamber. It is important to note that the rover model may need adjustments for different rover projects.

In order for the rover to execute the chosen global path on the lunar surface, it needs to be aware of the time epoch, direction, and location. Therefore, it is necessary to equip the rover with corresponding onboard sensors. Additionally, temperature and battery power will be utilized in posture control as described in Section 3.3.3. This research assumes that these sensors are standard for rover missions and readily available. The absence of sensor information may result in the rover being unable to accurately follow the selected global path.

It is also important to note that, in the proposed architecture, the map used for the training process and evaluation must be the same. In other words, a trained agent is not expected to work with an entirely new environment. While the agent is trained to perform efficiently in the presence of uncertainties within the selected map, it is not anticipated to function effectively in a completely different environment. This is due to variations in slope distributions across different maps, indicating that appropriate actions for a given state differ in distinct maps.

## 3. Model

### 3.1. Map Overview

Among many representation options for rough terrain, this research uses a two-and-a-half dimensional (2.5D) grid map for its efficiency in processing and data storage. The 2.5D grid map is represented as a collection of terrain properties (e.g., height, slope) over a uniform grid, while the 3D (three dimensional) map is profiling of objects in three dimensions to map the objects in the real world. This research implemented a grid map with information on slope angles.

**Figure 2:**
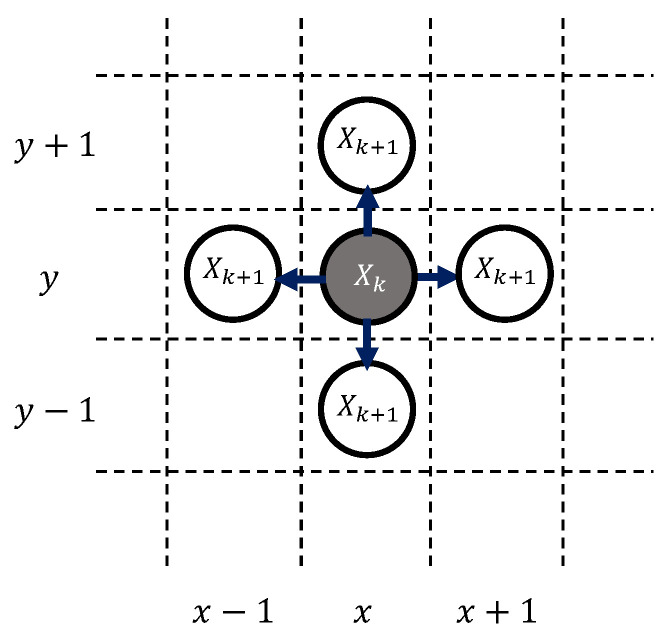
Rover motion model.

A path planning problem was solved over a generated grid map where the rover starts from an initial node of $X_{0}$ traveling incrementally to a goal node of $X_{f}$ while generating a path Ψ. This path is generated in a time sequential manner from {$X_{0}$, …, $X_{k}$, …, $X_{f}$}. The rover’s position $X_{k}$ is defined by unique grid coordinates corresponding a two-dimensional grid position, e.g., (*x*,*y*). The rover can move in any of the four directions on the map to node $X_{k+1}$, as shown in Figure 2. It is important to note that action *u* also includes *stay* action, which allows the rover to stay in the same grid position for one time step.

It is also important to note that this research also considers rover motion transition probability during the path planning. The transition probability for rover motion can be represented by $P_{a}$ in an MDP framework. In this research, the transition probability $P_{a}$ was defined such that the rover relocates to the planned location with a probability of $p_{t}$, regardless of state *s*. Alternatively, it may take another random action, including relocation to an unplanned location or staying in the same grid, with a probability of $1-p_{t}$.

This work used a 5 m resolution digital elevation model (DEM) based on a data product of the *Lunar Orbiter Laser Altimeter* (*LOLA*) instrument [24]. Based on the DEM, a grid-based map was implemented with one grid corresponding to 5 m. The height data of the DEM was used to compute a local slope angle and normal vector of the lunar surface at each grid point.

### 3.2. Lunar Environment

#### 3.2.1. Sun Vector

Due to the Moon’s synodic period averaging around 708 h, a singular lunar day corresponds to approximately 29.5 Earth days [25]. In regions with non-polar latitudes, this results in alternating cycles of daylight and darkness, each lasting an average of 14.75 Earth days. Sun vector is determined by the Sun’s position as viewed from a rover local latitude and longitude on the Moon at the moment of interest by using vector math.

#### 3.2.2. Lunar Surface Temperature

The thermal conditions on the lunar surface are extremely challenging. The absence of an atmosphere, combined with low surface conductivity and high emissivity, results in temperature fluctuations spanning from 100 K to 380 K [26]. According to [11], when the heat input from sunlight, i.e., $Q_{sun,m}$, and radiation heat transfer from the lunar surface to outer space, i.e., $Q_{sp,m}$, are balanced, the following equation was obtained:

(1) $$Q_{sun,m}=Q_{sp,m}$$

where $Q_{sun,m}$ and $Q_{sp,m}$ can be modeled as

(2) $$Q_{sun,m}\left(X_{k},t_{k}\right)=\alpha_{m}F_{sun,m}A_{m}D$$

(3) $$Q_{sp,m}\left(X_{k},t_{k}\right)=\sigma \epsilon_{m}F_{sp,m}A_{m}\left({T_{m}}^{4}-{T_{sp}}^{4}\right)$$

α is the absorptivity of the corresponding node, ϵ is the emissivity of the corresponding node, $F_{a,b}$ is the view factor from node *a* to node *b*, *A* is the surface area of the corresponding node, *D* is the solar irradiance constant, σ is the Stefan–Boltzmann constant, *T* is the temperature of the corresponding node, ${\;\cdot \;}_{m}$ indicates that the parameter is regarding the Moon (lunar surface) node, ${\;\cdot \;}_{sun}$ indicates that the parameter is regarding the Sun node, ${\;\cdot \;}_{sp}$ indicates that the parameter is regarding the outer space node.

Equations (1)–(3) can be solved with respect to the lunar surface temperature $T_{m}$ as shown in (4), and provides a reasonably approximated lunar surface temperature at lunar daytime (not valid for night-time) under the assumption that the lunar surface is composed of low-conductive material (i.e., regolith) and internal conductive heat transfer within the lunar soil can be ignored.

(4) $$T_{m}\left(X_{k},t_{k}\right)=\sqrt[{4}]{\frac{\alpha_{m}F_{sun,m}D+\sigma \epsilon_{m}F_{sp,m}{T_{sp}}^{4}}{\sigma \epsilon_{m}F_{sp,m}}}$$

As indicated in (4), lunar surface temperature changes depending on the view factor from the lunar surface to the sun, i.e., $F_{sun,m}$.

### 3.3. Rover Model

#### 3.3.1. Thermal Model

This research employed a thermal node model proposed by [11] for rover temperature prediction. The effectiveness of the proposed architecture is dependent on the accuracy of the rover model. Ref. [11] presents a thermal model used in the implementation of a real flight project [23], which has been calibrated through a thermal balance test conducted in a vacuum chamber. It is important to note that different rover projects may necessitate the use of a different thermal model.

In our definition, $Q_{sun}$ is the solar radiation from the sun, $Q_{a}$ is the surface albedo effect, $Q_{r}$ is the radiative heat transfer, $Q_{c}$ is the conductive heat transfer, $Q_{e}$ is the dissipated energy from on-board electronics or absorbed energy through solar power generation, and $Q_{sp}$ is the radiation emitted to outer space. Assuming that each rover surface has a specific nodal point, the following relation is derived from the first law of thermodynamics at a time $t_{k}$:

(5) $$M_{i}c_{p\;i}\frac{dT_{i}}{dt}=Q_{sun,i}+Q_{a,i}+Q_{r\;m,i}+Q_{c\;m,i}+Q_{sp,i}+Q_{e,i}+Q_{r\;i,j}+Q_{c\;i,j}$$

where *i* and *j* represent *i*-th and *j*-th surface node, $M_{i}$ is the mass of the *i*-th node, and $c_{p\;i}$ is the specific heat of the *i*-th node. Each heat transfer component is defined by the following equations:

(6) $$\begin{array}{cc} Q_{sun,i}\left(X_{k},t_{k},\theta_{k}\right) & =\alpha_{i}F_{sun,i}A_{i}D-W_{gen\;i} \end{array}$$

(7) $$\begin{array}{cc} Q_{a,i}\left(X_{k},t_{k},\theta_{k}\right) & =\alpha_{i}F_{m,i}A_{i}\left(1-\alpha_{m}\right)Q_{sun,m} \end{array}$$

(8) $$\begin{array}{cc} Q_{r\;m,i}\left(X_{k},t_{k}\right) & =\sigma \epsilon_{m}\epsilon_{i}F_{m,i}A_{i}\left({T_{m}}^{4}-{T_{i}}^{4}\right) \end{array}$$

(9) $$\begin{array}{cc} Q_{c\;m,i}\left(X_{k},t_{k}\right) & =k_{m,i}A_{c\;m,i}\left(T_{m}-T_{i}\right) \end{array}$$

(10) $$\begin{array}{cc} Q_{sp,i}\left(X_{k},t_{k}\right) & =\epsilon_{i}F_{sp,i}A_{i}\sigma \left({T_{sp}}^{4}-{T_{i}}^{4}\right) \end{array}$$

(11) $$\begin{array}{cc} Q_{e,i}\left(t_{k}\right) & =W_{i} \end{array}$$

(12) $$\begin{array}{cc} Q_{r\;i,j}\left(t_{k}\right) & =\sigma \epsilon_{i}\epsilon_{j}F_{i,j}A_{i}\left({T_{j}}^{4}-{T_{i}}^{4}\right) \end{array}$$

(13) $$\begin{array}{cc} Q_{c\;i,j}\left(t_{k}\right) & =k_{i,j}A_{c\;i,j}\left(T_{j}-T_{i}\right) \end{array}$$

where $A_{c\;a,b}$ is the contact area between the node *a* and node *b*, $k_{a,b}$ is the thermal contact conductance between the node *a* and node *b*, *W* is the electronics heat dissipation of the corresponding node, $W_{gen}$ is solar power generation of the corresponding node, which is only applicable to the surface covered by solar panels, and $\theta_{k}$ is the rover orientation at a time epoch $t_{k}$. It is important to note that the view factor from the *i*-th node to the sun, i.e., $F_{sun,i}$, changes depending on rover orientation $\theta_{k}$. This indicates that rover’s temperature (and power generation, as discussed in the next section) can be controlled by means of rover orientation control.

This analysis decomposed the rover into six thermal nodes, *Top*, *Right*, *Left*, *Front*, *Rear*, and *Bottom*. The temperature of each node, i.e., $T_{i}$, is calculated as

(14) $$T_{i}\left(\Psi_{k},\theta_{k}\right)\approx T_{i}\left(\Psi_{k-1},\theta_{k-1}\right)+\frac{dT_{i}}{dt}\;\cdot \;\Delta t$$

where Ψ is a rover path consisting of tuple of *X* and *t*, Δt is an interval of one time step. As indicated by (14), the rover temperature status $T_{i}$ is a path-dependent variable. In order to deal with the coupling heat transfer such as $Q_{r\;i,j}$ and $Q_{c\;i,j}$, the equations must be solved iteratively.

#### 3.3.2. Power Model

Power generation at a time $t_{k}$ can be modeled by the following equation:

(15) $$W_{gen\;i}\left(X_{k},t_{k},\theta_{k}\right)=p_{i}eQ_{sun,i}\left(X_{k},t_{k},\theta_{k}\right)$$

where $p_{i}$ is a ratio of area covered by solar cells in relation to the entire surface area of the node, *e* is the power conversion efficiency including solar cell efficiency and power conversion loss. $p_{i}$ is set 0 when the *i*-th surface is not equipped with solar cells. It is important to note that the power generation $W_{gen}$ can be controlled by means of rover orientation as $Q_{sun,i}$ is a function of $F_{s,i}$. Battery charging occurs when the generated power surpasses the total power consumption:

(16) $$W_{avail}=\sum_{i}W_{gen\;i}-\sum_{i}W_{i}$$

where $W_{avail}$ is available power for battery charging. When $W_{avail}$ is a negative value, the battery will be discharged. The remaining battery power *B* changes over time according to the following equation:

(17) $$B\left(\Psi_{k},\theta_{k}\right)=B\left(\Psi_{k-1},\theta_{k-1}\right)+W_{avail}\;\cdot \;\Delta t$$

Then, the percentage of remaining power $B_{\%}$ in relation to the maximum battery capacity $B_{max}$ is calculated by:

(18) $$B_{\%}\left(\Psi_{k},\theta_{k}\right)=B\left(\Psi_{k},\theta_{k}\right)/\left(B_{max}\right)\;\cdot \;100$$

As indicated by (18), rover power status $B_{\%}$ is a path-dependent variable.

#### 3.3.3. Rover Orientation Control

As indicated by Equations (14) and (18), the resulting rover thermal and power status become different depending on the rover orientation. Therefore, rover orientation control must be considered in addition to the rover’s location and relocation timing during the path planning process. However, it will be computationally expensive if an additional parameter in the path search process is considered, for instance, by adding rover orientation to the state *s*.

Therefore, an optimal rover orientation control function was implemented within the rover thermal and power model module. The function was named *greedy orientation control*, as depicted in Figure 1.

In the rover system, the thermal model is not linear with respect to the rover orientation. Therefore, the conventional linear feedback approach such as proportional–integral–derivative controller or linear quadratic regulator cannot be used to find an optimal rover orientation. In this context, an exhaustive search algorithm was utilized to determine the optimal rover orientation $\theta^{*}$ that minimizes the sum of designed thermal and power penalty function *C*:

(19) $$\begin{array}{c} \min_{\theta }C\left(t_{k},\theta \right)\\ C\left(t_{k},\theta \right)=pe_{thermal}\left(t_{k},\theta \right)+pe_{power}\left(t_{k},\theta \right) \end{array}$$

where $pe_{thermal}$ and $pe_{power}$ are thermal penalty and power penalty. In this function, an optimal rover orientation is chosen with regard to the designed thermal and power penalty function. The mathematical models of thermal and power penalty function are described in detail in Section 3.4.

Consequently, Equations (14) and (18) can be expressed with using a uniquely determined optimal rover orientation $\theta^{*}$ as

(20) $$T_{i}\left(\Psi_{k},\theta_{k}^{*}\right)\approx T_{i}\left(\Psi_{k-1},\theta_{k-1}^{*}\right)+\frac{dT_{i}}{dt}\;\cdot \;\Delta t$$

(21) $$B_{\%}\left(\Psi_{k},\theta_{k}^{*}\right)=B\left(\Psi_{k},\theta_{k-1}^{*}\right)/\left(B_{max}\right)\;\cdot \;100$$

In actual missions, it is realistic to assume that the rover is equipped with some sort of on-board orientation control algorithm, enabling the implementation of the proposed orientation control algorithm.

### 3.4. Rewards for Training

A reward function is used for the reinforcement learning process. A reward function that consists of time penalty $pe_{time}$, terramechanical penalty $pe_{slope}$, thermal penalty $pe_{thermal}$, power penalty $pe_{power}$, positioning reward $re_{pos}$, and goal reward $re_{goal}$ was designed:

(22) $$Re\left(t_{k}\right)=-pe_{time}-pe_{slope}-pe_{thermal}-pe_{power}+re_{pos}+re_{goal}$$

#### 3.4.1. Time Penalty

A fixed-value time-penalty tp was utilized for each step:

(23) $$pe_{time}=tp$$

#### 3.4.2. Terramechanical Penalty

A terramechanical penalty is calculated based on the slope value of the rover position as

(24) $$pe_{slope}=\left\{\begin{array}{cc} K_{s}\;\cdot \;Sl{\left(X_{k}\right)}^{2}, & \mathrm{if}\;|Sl\left(X_{k}\right)|>sl_{th}\\ K_{s}\;\cdot \;Sl{\left(X_{k}\right)}^{2}+E_{s}, & \mathrm{otherwise} \end{array}\right.$$

where Sl(·) returns a slope angle of the corresponding grid $X_{k}$, $K_{s}$ is a scaling factor, $sl_{th}$ is a user-set slope angle threshold, and $E_{s}$ is an extra penalty which is only applied when the slope exceeds the maximum traversable slope angle $sl_{th}$.

#### 3.4.3. Thermal Penalty

The goal of thermal systems design is to keep all of the electronics components within their operating temperature thresholds, i.e., $T_{min}$ and $T_{max}$, while the rover traveling over the selected path. Accordingly, the success criterion of thermal control at a time $t_{k}$ is determined by how well and whether the temperature of electronics is maintained within the thermal safety thresholds. In our model, the temperature of the *Top* surface represents the temperature of electronics, assuming that majority of the electronics boards are mounted on the *Top* surface according to the micro-rover thermal design proposed by [23].

As shown in (25), the thermal penalty function is designed using a power function with the power factor $e_{t}$, which increases the penalty exponentially based on the difference between the user-set control target $T_{c}$ and the *Top* surface’s temperature $T_{top}$, and the designed threshold $T_{th}$. $K_{t}$ is a scaling factor.

(25) $$pe_{thermal}=K_{t}\;\cdot \;{\left(\frac{\left|T_{c}-T_{top}\left(\Psi_{k},\theta_{k}\right)\right|}{T_{th}}\right)}^{e_{t}}$$

(26) $$T_{c}=\left\{\begin{array}{cc} T_{min}, & \mathrm{if}\;T_{top}>\left(T_{min}+T_{max}\right)/2\\ T_{max}, & otherwise \end{array}\right.$$

It is important to note that $pe_{thermal}$ is a function of path $\Psi_{k}$ and orientation $\theta_{k}$. However, $\theta_{k}$ is optimized and removed by the greedy orientation control, as mentioned earlier.

#### 3.4.4. Power Penalty

Power management safety is determined by the battery depth of discharge (DoD). A battery’s life is affected by the number of charge/discharge cycles, so a low DoD contributes to the longevity of the battery. Accordingly, the success criterion of power control at a time $t_{k}$ is determined by how well and whether the percentage of the remaining battery power is kept beyond a certain threshold $B_{\%\;min}$.

Similarly to the thermal penalty function, the power penalty function is designed using a power function with the power factor $e_{p}$, which increases the penalty exponentially based on the difference between the user-set control target $B_{\%\;c}$ and current remaining power percentage $B_{\%}$, and the designed threshold $B_{\%\;th}$. $K_{p}$ is a scaling factor.

(27) $$pe_{power}=K_{p}\;\cdot \;{\left(\frac{\left|B_{\%\;c}-B_{\%}\left(\Psi_{k},\theta_{k}\right)\right|}{B_{\%\;th}}\right)}^{e_{p}}$$

$B_{\%\;c}$ is usually set high enough. As with the thermal penalty, $pe_{power}$ is a function of path $\Psi_{k}$ and orientation $\theta_{k}$. However, $\theta_{k}$ is optimized and removed by the greedy orientation control.

#### 3.4.5. Positioning Reward

Position reward is calculated based on an Euclidean distance obtained at a time epoch $t_{k}$. It is designed to encourage the rover to approach the goal node, i.e., the closer the rover gets to the goal node, the more reward will be awarded by the end of the episode.

(28) $$re_{pos}=K_{pos}\;\cdot \;\left(\left|\right|X_{f}-X_{k-1}\left|\right|-\left|\right|X_{f}-X_{k}\left|\right|\right)$$

#### 3.4.6. Goal Reward

The goal reward is provided by the following equation:

(29) $$re_{goal}=\left\{\begin{array}{cc} g, & \mathrm{if}\;X_{k}\;\mathrm{corresponds}\;\mathrm{to}\;\mathrm{the}\;\mathrm{goal}\;\mathrm{grid}\;X_{f}\\ 0, & \mathrm{otherwise}\; \end{array}\right.$$

where *g* is a goal reward which is given only when $X_{k}$ corresponds to the goal grid $X_{f}$.

## 4. Numerical Results and Analysis

### 4.1. Implementation Details

In this study, the simulation environment was constructed using *MiniGrid* [27]. MiniGrid is an open-source general grid environment that is compatible with the *OpenAI Gym platform* [28]. It necessitates customization based on user scenarios, and in our case, it was tailored for a lunar rover exploration scenario.

Next, the environment model was integrated into the grid map simulation. Initially, each grid was assigned a slope value extracted from the 5 m resolution LOLA DEM of a location of interest. Subsequently, the sun vector and lunar surface temperature for the entire mission period were calculated for each grid using a fixed time step before initiating the path search process. While environmental factors (slope, sun vector, and lunar surface temperature) can be populated prior to the path search, the rover’s thermal and power status are path-dependent and thus need to be calculated during the path search.

Time step interval Δt (which is essentially how long it takes for the rover to take one action on the Moon) was fixed to 30 min. The initial time epoch was set to approximately 75 h before local noon in order to create a challenging lunar thermal environment, where the rover will experience a temperature increase at the beginning, reach its highest temperature at noon, and then experience a temperature decrease as it approaches evening/night.

As for the implementations of the reinforcement learning algorithm, open source software code called *Stable Baselines3* [29] was utilized. Tuning of learning hyperparameters plays a large role in eliciting the best results from learning algorithms. For instance, [30] demonstrated the effects of specific hyperparameters on algorithm performance. In this research, the choice of particular hyperparameters significantly influences both the training efficiency and the subsequent performance of the trained agent. Therefore, they need to be chosen carefully. In this regard, this research used the exhaustive grid search method, which is currently the most widely used method, for parameter optimization [31]. Table 2 summarizes the hyperparameters used in our simulation. These hyperparameters were defined in accordance with Stable Baselines3 standard definition. If hyperparameters are not specified in the table, the default value used in Stable Baselines3 were used. The same hyperparameters were used throughout all the simulations.

The learning agent was then trained for a 100 × 100 grid map. The initial node was set as $X_{0}=\left(5,5\right)$, and the goal node was set as $X_{f}=\left(95,95\right)$. Table 3 summarizes the designed reward parameters. The same parameters were used throughout all the simulations. After the training, the acquired learning agent was applied to a new episode for evaluation.

### 4.2. Simulation Results

To test the applicability of the proposed architecture in various settings, the performance of the architecture was evaluated with two different maps. Two maps were created based on the lunar DEM of $45^{{}^{\circ}}$ latitude and $0^{{}^{\circ}}$ longitude, with a slight difference in location of approximately 2 km, which were labeled as Scenario 1 and Scenario 2.

**Figure 3:**
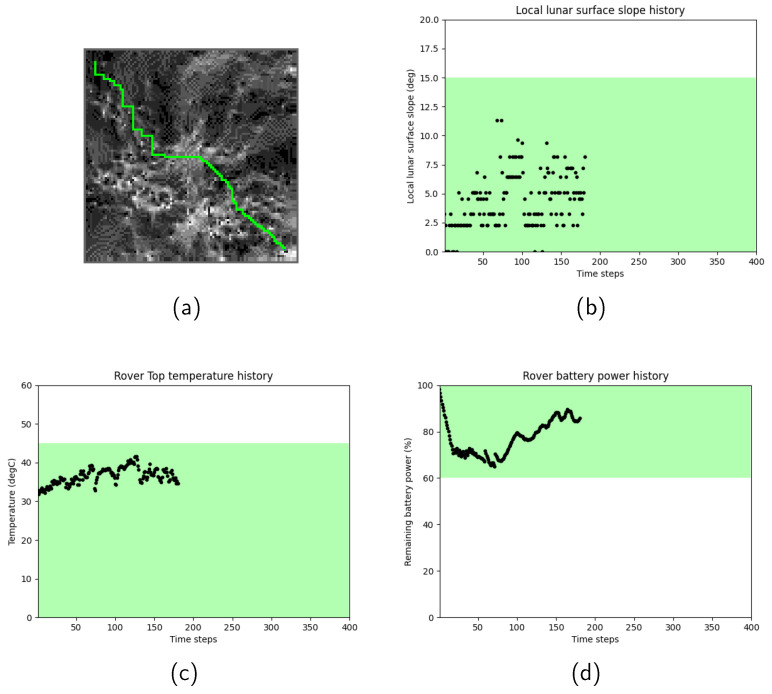
Scenario 1 path search results: (**a**) the selected rover path over the terrain map, (**b**) history of the slope angles, (**c**) history of the rover’s top temperature, (**d**) history of the rover’s battery power.

Figure 3 shows the path search results for Scenario 1, when the rover motion transition probability $p_{t}$ was set to 0. In Figure 3, (a) the selected rover path shown in light-green color starts from the initial node on top left and moves toward the goal node on right bottom, where slope values are expressed in gray scale. Dark grids correspond to gentle slopes, whereas bright grids correspond to steep slopes. It was observed that the rover successfully chose a path by avoiding grids that have a large slope. The history of the slope angles (b), rover’s temperature (c), and remaining battery power (d) were also plotted, respectively. In each figure, the designed safety range is highlighted in green. It was confirmed that the selected path satisfied the terramechanical, thermal, and power safety ranges at all data points.

**Figure 4:**
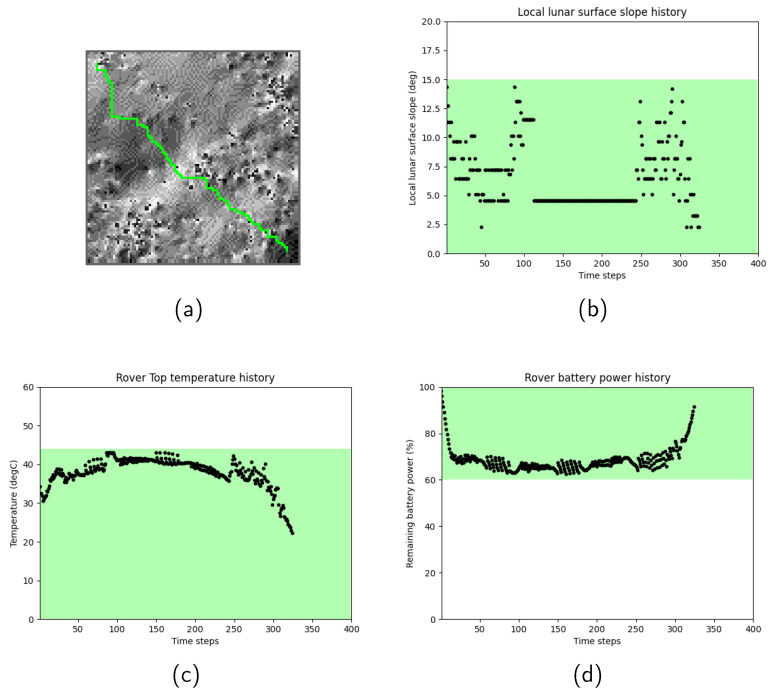
Scenario 2 path search results: (**a**) the selected rover path over the terrain map, (**b**) history of the slope angles, (**c**) history of the rover’s top temperature, (**d**) history of the rover’s battery power.

**Figure 5:**
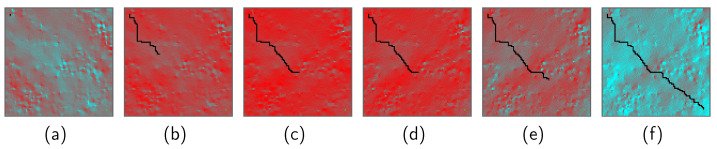
Selected rover path over the lunar surface temperature maps. Each figure corresponds to a different time epoch. (**a**) after 2 steps, (**b**) after 65 steps, (**c**) after 130 steps, (**d**) after 195 steps, (**e**) after 230 steps, (**f**) after 318 steps (arriving at the goal node).

Figure 4 show the path search results for Scenario 2. In Scenario 2, due to the more challenging lunar surface environment, characterized by higher lunar surface temperatures and more undulating terrain, the total time epoch of the selected path became longer. To further investigate the history of the rover’s motion, the relationship between the lunar surface temperature profile and the selected path were examined. Figure 5 shows the path search result over the lunar surface temperature map. In these figures, grids are colored based on their temperatures. Red grids correspond to high temperatures, whereas light-blue grids correspond to low temperatures. Black grids represent the selected rover path. It was observed that the rover took *stay* actions in the middle of the mission period, between Figure 5c and Figure 5d, in order to stay at relatively low-temperature grids until the path toward the goal node became thermally available.

### 4.3. Probabilistic Simulation

Next, the performance of the trained agents was evaluated with the rover motion transition probability to gain a deeper understanding of their capabilities.

The training process accounts for rover probabilistic motion influenced by the exploration rate. Hence, the trained agent is anticipated to develop resilience in dealing with uncertainties during the training process. During evaluation, the probabilistic rover motion simulates scenarios in which the rover must take unplanned actions due to unexpected reasons. Consequently, the results offer insights into the trained agent’s ability to withstand uncertainties.

Table 4 summarizes path search results with three selected rover motion transition probabilities, $p_{t}=0$, $p_{t}=0.02$, and $p_{t}=0.05$, for the two cases discussed in Section 4.2. Each data point represents the averaged performance of 10 different simulation runs using the same trained agent.

In some cases, the rover motion transition probability resulted in a violation of the safety criteria. Since constraints are treated as costs, the proposed algorithm does not guarantee the satisfaction of the safety criteria. An alternative approach involves terminating the episode as soon as any constraint is violated and imposing a very high penalty to enforce strict compliance with the safety criteria. We will investigate this in our future work.

In addition, Scenario 2 exhibited a larger performance variance, resulting in more violations of safety criteria. This outcome suggests that addressing Scenario 2 effectively within the context of probabilistic rover motion may be challenging. It is suspected that the range of paths free from violations is narrower and more prone to infringement when the transition probability of rover motion is higher in Scenario 2. In essence, the proposed architecture successfully demonstrated the sensitivity of path search to unforeseen uncertainties.

## 5. Comparative Analysis

In this section, a comparative analysis will be conducted to underscore the advantages of *the rover resource-based path search* over *the environment-based path search*, an aspect that has not been explored in existing research.

As mentioned in Section 1, the existing research relies solely on environmental constraints, such as terramechanics, luminous, and thermal constraints, as analyzed in [7,8,9]. Consequently, a map encompasses both static and time-variant constraints. Therefore, methods designed to handle only static constraints, such as Dijkstra, are not directly applicable to the generated map. Moreover, A* is inefficient for solving maps with time-variant constraints, since it requires the map to be extended in the time-direction, imposing a significant computational burden on the solver. Instead, this research proposes emulating the environment-based path search within the proposed framework by adjusting both the state *s* and the reward function Re. We believe this is a straightforward yet valid approach to confirming the advantages of resource-based path search over the environment-based path search.

### 5.1. Environment-Based Path Search

In the environment-based path search, rover temperature will be controlled based on environmental conditions by adding a high penalty to extreme lunar surface temperature. For this purpose, a new penalty function $pe_{env}$ was defined as

(30) $$pe_{env}\left(t_{k}\right)=\left\{\begin{array}{cc} K_{e}\;\cdot \;|T_{m\;c}-T_{m}\left(X_{k},t_{k}\right)|, & \mathrm{if}\;T_{m\;min}<T_{m}\;\mathrm{and}\;T_{m}<T_{m\;max}\\ K_{e}\;\cdot \;|T_{m\;c}-T_{m}\left(X_{k},t_{k}\right)|+E_{e} & \mathrm{otherwise} \end{array}\right.$$

(31) $$T_{m\;c}=\left\{\begin{array}{cc} T_{m\;min}, & \mathrm{if}\;T_{m}>\left(T_{m\;min}+T_{m\;max}\right)/2\\ T_{m\;max}, & otherwise \end{array}\right.$$

(32) $$E_{e}=\left\{\begin{array}{cc} K_{e2}\;\cdot \;\left(T_{m}\left(X_{k},t_{k}\right)-T_{m\;max}\right), & \mathrm{if}\;T_{m}>T_{m\;max}\\ K_{e2}\;\cdot \;\left(T_{m\;min}-T_{m}\left(X_{k},t_{k}\right)\right), & \mathrm{if}\;T_{m\;min}>T_{m} \end{array}\right.$$

where $T_{m}$ is a lunar surface temperature of the rover location, $T_{m\;c}$ is a control target, which is chosen either from $T_{m\;min}$ or $T_{m\;max}$, depending on which is closer to the current lunar surface temperature, and $K_{e}$ and $K_{e2}$ are user-set scaling factors. The designed penalty consists of two elements, $K_{e}\;\cdot \;|T_{m\;c}-T_{m}\left(X_{k},t_{k}\right)|$ and $E_{e}$, where the first element is given proportional to how much a lunar surface temperature of the new location deviates from the control target $T_{m\;c}$, and the second element is an extra penalty which is only applied when a lunar surface temperature of the rover location exceeds the target range.

Penalty function $pe_{env}$ can also contribute to the rover power status control. As the designed rover only has solar arrays on its side panels (i.e., *Right* and *Left*), low power generation occurs when the sun inclination angle is high, which is equivalently when the lunar surface temperature is high. Therefore, poor luminous conditions can be avoided by avoiding extremely high lunar surface temperature.

As a result, overall reward function for the environment-based path search is defined by updating (22) to:

(33) $$Re\left(t_{k}\right)=-pe_{time}-pe_{slope}-pe_{env}+re_{pos}+re_{goal}$$

where other penalty and reward functions, including $pe_{time}$, $pe_{slope}$, $re_{pos}$, and $re_{goal}$, remain the same. Also, the state *s* was modified to only accommodate rover position $X_{k}$, time epoch $t_{k}$, and lunar surface temperature of the rover location $T_{m}$.

Finally, training was performed over the same map according to the updated state *s* and reward function Re. The selected reward design parameters are summarized in Table 5. The same learning hyperparameters in Table 2 were used.

### 5.2. Comparison Results

**Figure 6:**
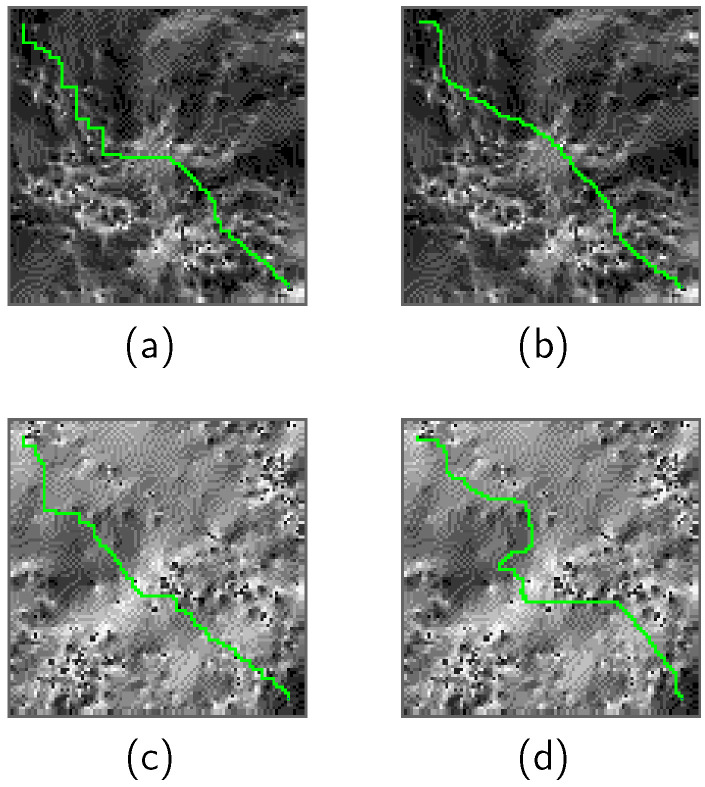
Comparison between the rover resource-based path search and the environment-based path search. Figures (**a**–**d**) depict the selected rover paths for different search methods and scenarios, where (**a**) corresponds to the rover resource-based path search in Scenario 1, (**b**) corresponds to the environment-based path search in Scenario 1, (**c**) corresponds to the rover resource-based path search in Scenario 2, and (**d**) corresponds to the environment-based path search in Scenario 2. (**a**) is a duplicate of Figure 3a, while (**c**) is a duplicate of Figure 4a.

Figure 6 is the comparison between the rover resource-based path search and the environment-based path search. Table 6 displays a quantitative comparison of the performance. It is crucial to note that the reward designs differ, implying that a direct comparison of reward values between the two path search methods is not meaningful.

In Scenario 1, the distinction between the rover resource-based path search and the environment-based path search is not significant, as both exhibit similar performance in terms of total time steps and violation of safety criteria. In contrast, the rover resource-based path search outperformed the environment-based path search in Scenario 2, with an increased number of total time steps in the environment-based path compared to the rover resource-based path search. This result suggests that the rover utilized its thermal and power capacitance to temporarily navigate through excessively challenging environmental conditions encountered in Scenario 2, indicating that the rover resource-based path search has the potential to generate better-optimized paths.

It is worth mentioning again that existing approaches, such as A*, cannot be used to address path-dependent constraints, and should be considered limited compared to the rover resource-based path search conducted in this research, particularly in terms of the variety of constraints that can be considered. A* is capable of handling time-variant constraints by extending the map in the time dimension. Therefore, A* can perform competitively with the environment-based path search conducted in this research.

## 6. Discussions

### 6.1. Reproducibility of the Training Results

Due to random variables introduced in the proposed architecture, such as the rover’s random actions influenced by the exploration rate, trained agents exhibited variances in performance. This not only impacts performance evaluation, as mentioned in Section 4.3, but also influences the training process itself.

**Figure 7:**
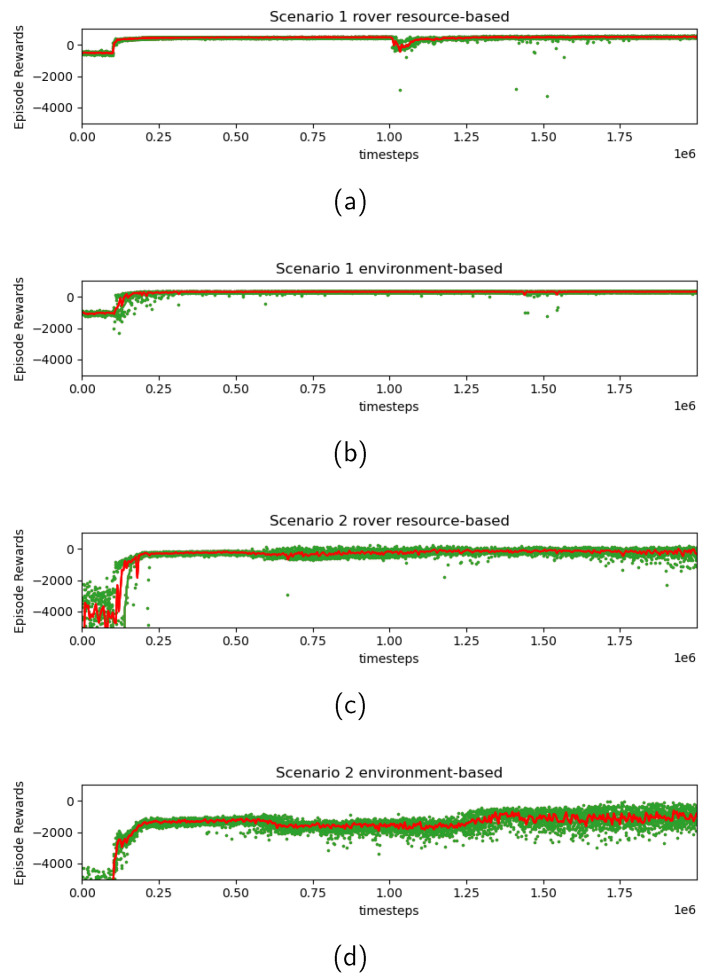
Reward transition during training processes in various scenarios. (**a**) corresponds to the rover resource-based path search in Scenario 1, (**b**) corresponds to the environment-based path search in Scenario 1, (**c**) corresponds to the rover resource-based path search in Scenario 2, and (**d**) corresponds to the environment-based path search in Scenario 2.

Figure 7 depicts the transition of received rewards during the entire training process. The green dots represent rewards received at the end of each episode, while the red line depicts a moving average taken over every 5000 time steps. The training profile indicates that low reward values (in other words, large penalties) observed initially have successfully converged to better values through the training process. Additionally, in Scenario 2, greater variance was observed at the end of training compared to Scenario 1, in both rover resource-based and environment-based searches. As mentioned in Section 4.3, the higher complexity of the Scenario 2 environment contributed to this increase in variance.

For practical applications, additional refinement of rewards, adjustment of hyperparameters, or the adoption of more sophisticated learning algorithms could mitigate the variance in path search results. An alternative strategy involves terminating the episode during training promptly upon any constraint violation, and imposing a very high penalty, which is deemed valuable to enhance constraint satisfaction. These aspects will be explored further in our future work.

### 6.2. Reward Tuning

The design of the reward function has an impact on the resulting path selection. For instance, the balance of scaling factors, namely $K_{s}$, $K_{t}$, and $K_{p}$, affects which constraints must be prioritized in consideration for the orientation control and path search.

**Figure 8:**
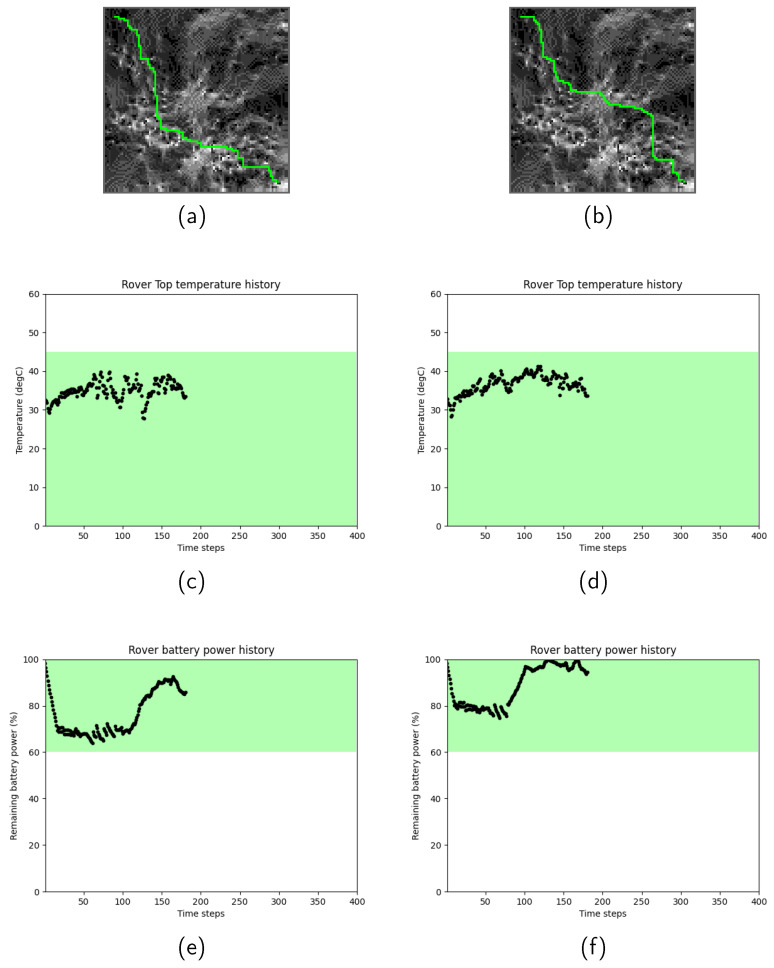
Path search results with different combinations of $K_{t}$ and $K_{p}$. (**a**,**c**,**e**) represent the case with $K_{t}=4$ and $K_{p}=2$ (i.e., thermal prioritized), while (**b**,**d**,**f**) represent the case with $K_{t}=2$ and $K_{p}=20$ (i.e., power prioritized).

As an example, Figure 8 depicts the path selection and the resulting history of the rover’s resource status in the rover resource-based path search in Scenario 1. Two different combinations of scaling factors are presented: (a), (c), and (e) represent the case with $K_{t}=4$ and $K_{p}=2$ (i.e., thermal prioritized), while (b), (d), and (f) represent the case with $K_{t}=2$ and $K_{p}=20$ (i.e., power prioritized). The values of $K_{t}$ and $K_{p}$ were chosen to be sufficiently distinct to yield noticeably different results, while an excessively extreme value can lead to inefficient path selections. The thermal and power profiles of the generated paths exhibited intriguing characteristics; one showed a superior power history compared to the other, while both thermal histories had minor differences. This result suggests that improving the rover’s thermal status is more challenging, even when sacrificing the power profile, under the selected conditions. This example effectively demonstrates how the proposed method can enhance our understanding of the interrelationships among the constraints and path selection.

### 6.3. Map Size and Computation Time

In an assumed mission scenario, calculations are executed in a mission operation center prior to a mission, and the results will be utilized either by ground operators for manual operation or uploaded to space rovers through telecommands for autonomous operation. Consequently, the proposed system is not constrained by the performance of on-board rover computation. However, in scenarios where mission re-planning is necessary recursively, the intensive computation required may introduce delays in mission operations. This concern is anticipated to be addressed in the future, given the potential availability of high-performance cloud-based computing services to the public within a few years.

### 6.4. More Assumptions for Realistic Missions

In this research, rover motion was constrained to four cardinal directions and stationary actions. In a more realistic scenario, diagonal motion could prove beneficial for shortening the travel path. Additionally, the paper assumed a uniform time for the rover to move across a cell. However, the time required for cell traversal depends on factors such as slope, friction, and battery level. Thus, these conditions should be considered when calculating the travel time at each cell. In the current framework, the values of sun vectors and lunar surface temperature for the entire mission period are precalculated with a fixed time step to reduce computational time during path search, as described in Section 4.1. However, this approach cannot be applied if the traveling times for each grid/action are different, leading to a significant increase in computational time. While anticipated advancements in computer science technology are expected to alleviate the computational load, we acknowledge the necessity of addressing this aspect in our future work.

### 6.5. Potential Application

When traveling in shadowed regions on Moon, thermal and power resource management becomes more constrained. The rover will explore shadowed regions by alternately traveling in illuminated and unilluminated regions, which induces more dynamic variations in thermal and power environmental conditions. In such a situation, a path search based on environment conditions may not work effectively, and the necessity of the rover resource-based path search is increased to improve the exploration range.

## 7. Conclusions

This research has introduced an innovative approach to global path and resource management planning for lunar rovers. Our proposed method incorporates static, time-variant, and path-dependent constraints into a grid map as a penalty function, utilizing a reinforcement learning framework to tackle a resource constrained shortest path problem.

To assess the performance of our proposed approach, lunar rover path search problems that encompass three distinct constraints (rover terramechanics performance, thermal status management, and power status management) were formulated. Subsequently, the proposed learning architecture was applied to these designed path search problems for evaluation. The simulation results demonstrate the effectiveness of our architecture in successfully identifying a rover path, while consistently meeting user-defined safety criteria related to terramechanical, thermal, and power considerations at all positions and time intervals. Additionally, through comparative analysis, it was verified that our proposed approach outperforms a conventional method that solely relies on static and time-variant constraints.

To enhance the performance of the proposed architecture in realistic mission scenarios, additional efforts need to be undertaken. Specifically, there is a need for the implementation of more flexible rover motion in terms of direction and length of the time step, along with an analysis of its impact on computational time.

## Figures and Tables

**Table 1:** Comparison of global path planning approaches.

Items	Proposed Method	Dijkstra, A*
Type of constraints	Static, time-variant, and path-dependent	Static and time-variant ^1^
Type of managements	Environment-based and resource-based	Environment-based only
Motion transition probability	Yes	No

^1^ To cope with time-variant constraints using the A* algorithm, a time-variant environment must be represented by a 3D binary array composed of stacked 2D maps for each time step.

**Table 2:** Learning hyperparameters.

Item	Value
Training time steps	2,000,000
Learning rate	0.0002
Learning starts	100,000
Discount factor (gamma)	0.995
Soft update coefficient (tau)	0.1
Net architecture	[64 64 64 64]

**Table 3:** Reward design parameters.

Item	Symbol	Value	Unit
Time penalty	tp	0.01	-
Slope threshold	$Sl_{th}$	15	deg
Thermal threshold	$T_{th}$	40	degC
Power threshold	$B_{\%\;th}$	37	%
Power control target	$B_{\%\;c}$	100	%
Thermal exponent	$e_{t}$	10	-
Power exponent	$e_{p}$	10	-
Scaling factor (position reward)	$K_{pos}$	5	-
Scaling factor (slope penalty)	$K_{s}$	0.01	-
Scaling factor (thermal penalty)	$K_{t}$	2	-
Scaling factor (power penalty)	$K_{p}$	2	-
Minimum operating temperature	$T_{min}$	0	degC
Maximum operating temperature	$T_{max}$	45	degC
Minimum operating battery power	$B_{\%\;min}$	60	%
Extra penalty (slope penalty)	$E_{s}$	20	-
Goal reward	*g*	100	-

**Table 4:** Simulation results with the rover motion transition probability.

Scenario	Motion Probability $p_{t}$	Total Time Steps Ave.	# of Thermal Violation Ave.	# of Power Violation Ave.	# of Slope Violation Ave.	Reward Ave.
1	0	180	0	0	0	469.9
	0.02	184.0	0	0	0	466.3
	0.05	188.1	0	0	0	457.3
2	0	318	0	0	0	−29.5
	0.02	318.8	2.0	0	1.1	−66.7
	0.05	319.3	7.0	0.4	1.8	−155.5

# of thermal/power/slope violation: a number of time steps violating the minimum operating temperature $T_{min}$ or maximum operating temperature $T_{max}$, or the minimum operating battery power $B_{\%\;min}$, or the slope threshold $Sl_{th}$, respectively.

**Table 5:** Reward design parameters for the environment-based case.

Item	Symbol	Value	Unit
Time penalty	tp	0.01	-
Slope threshold	$Sl_{th}$	15	deg
Scaling factor (position reward)	$K_{pos}$	5	-
Scaling factor (slope penalty)	$K_{s}$	0.01	-
Scaling factor (environment)	$K_{e}$	0.025	-
	$K_{e2}$	10	-
Minimum lunar surface temperature	$T_{m\;min}$	0	degC
Maximum lunar surface temperature	$T_{m\;max}$	85	degC
Extra penalty (slope penalty)	$E_{s}$	20	-
Goal reward	*g*	100	-

**Table 6:** Comparative analysis with the rover motion transition probability.

Environment-Based Path Search						
**Scenario**	**Motion Probability** $p_{t}$	**Total Time Steps Ave.**	# **of Thermal Violation Ave.**	# **of Power Violation Ave.**	# **of Slope Violation Ave.**	**Reward Ave.**
1	0.0	180	0	0	0	335.4
	0.02	181.4	0	0	0.2	328.9
	0.05	185.3	0	0	0	326.2
2	0.0	364	0	0	0	−13.2
	0.02	365.6	0	0	2	−87.9
	0.05	362.6	0.5	0	4.1	−224.9
Resource-Based Path Search *						
**Scenario**	**Motion Probability** $p_{t}$	**Total Time Steps Ave.**	# **of Thermal Violation Ave.**	# **of Power Violation Ave.**	# **of Slope Violation Ave.**	**Reward Ave.**
1	0	180	0	0	0	469.9
	0.02	184.0	0	0	0	466.3
	0.05	188.1	0	0	0	457.3
2	0	318	0	0	0	−29.5
	0.02	318.8	2.0	0	1.1	−66.7
	0.05	319.3	7.0	0.4	1.8	−155.5

* Resource-based search is a duplicate of Table 4.

## Data Availability

Data are contained within the article.
